# Macromolecular Characterization of Swordfish Oocytes by FTIR Imaging Spectroscopy

**DOI:** 10.1038/s41598-019-45065-7

**Published:** 2019-06-20

**Authors:** Oliana Carnevali, Michela Candelma, Andrea Sagrati, Paolo Pignalosa, Elisabetta Giorgini, Giorgia Gioacchini

**Affiliations:** 1 Dipartimento Scienze della Vita e dell’Ambiente, Università Politecnica delle Marche, Via Brecce Bianche, 60131 Ancona, Italy; 2 OCEANIS srl, Via Marittima, 59, - 80056 ERCOLANO, NA Italy

**Keywords:** Oogenesis, Biophysical chemistry

## Abstract

During folliculogenesis, primary oocytes of teleosts grow by several orders of magnitude by-self synthesizing proteins and mRNA, or sequestering from blood specific macromolecular components, such as fatty acids and vitellogenin. All these materials are stored into cortical alveoli, yolk globules or oil droplets during oocyte development. The proper synthesis, storage and displacement of these macromolecular components inside the oocyte play a key role for a successful fertilization process and for the subsequently correct embryo development. In this study, for the first time, the FTIR Imaging (FTIRI) spectroscopy has been applied to characterize the chemical building blocks of several cellular components of swordfish oocytes at different developmental stages. In particular, the spectral features of previtellogenic (PV), vitellogenic (VTG), mature (M) and atretic (A) follicles as well as and of cortical alveoli (CA), yolk vesicles (YV), oil droplets (OD) and Zona Radiata (ZR) have been outlined, providing new insights in terms of composition and topographical distribution of macromolecules of biological interest such as lipids, proteins, carbohydrates and phosphates. The macromolecular characterization of swordfish oocytes at different developmental stages represents a starting point and a useful tool for the assessment of swordfish egg quality caught in different conditions, such as periods of the year or different fishing area.

## Introduction

In teleost fish, eggs are the final product of oogenesis, a process during which the oocyte growths and differentiates. Eggs need all the necessary information to direct the development of free-swimming larvae as well all the ‘building blocks’ such as amino acids, lipids, carbohydrates and maternal determinants, to form a viable embryo^1^. These components are produced by the oocyte it-self or derived from several maternal sources and incorporated into the oocyte during oogenesis. When eggs lack specific compounds, or contain inappropriate amounts of them, they will be not able to sustain the development of a viable embryo. Hence, egg’s quality is strictly related to its cytoplasm composition^2,3^.

Mediterranean Swordfish (*Xiphias gladius*) is a large, highly migratory and valuable commercial species, which has been recently put through a stock recovery plan by the International Commission for the Conservation of Atlantic Tunas (ICCAT). Despite this, until now, information on its reproduction is scarce^4,5^, even if a deep knowledge on its reproductive potential is mandatory for studies on stock assessment and management. In this light, the objective of the present study was to characterize the biochemical composition and structure of swordfish oocytes at different developmental stages. At this purpose, previtellogenic, vitellogenic, mature and atretic follicles retrieved from individuals caught in the central Mediterranean Sea, were analysed for the first time, by Fourier Transform Infrared Imaging (FTIRI) spectroscopy and their macromolecular fingerprint defined.

In particular, the spectral features of oocytes at different developmental stage and of cortical alveoli (CA), yolk vesicles (YV), oil droplets (OD) and Zona Radiata (ZR) have been outlined, providing new insights in terms of composition and topographical distribution of macromolecules of biological interest such as lipids, proteins, carbohydrates and phosphates.

FTIRI is a well-established technique for the analysis of the macromolecular building blocks of cells and tissues^6^. By coupling IR spectrometers with bidimensional arrays detectors, it is possible to spectroscopically map specific areas of non-homogeneous biological samples, providing, at the same time and on the same sample, unique biochemical and ultrastructural information^7,8^. In recent years, several reports exploited FTIRI to characterize the biochemical changes associated with oocyte growth and maturation, in various species including fish^9–13^. In addition, recently, this spectroscopic tool has been also applied to evaluate the macromolecular alterations induced in zebrafish by feed additives and pollutants^14–16^.

Advances in comprehensive understanding of oogenesis process obtained by FTIRI will undoubtedly contribute to improve knowledge on the effects of environment (pollutants, food, overfishing, etc.) on egg quality and integrity in a wild and endangered species such as swordfish. By understanding the molecular and morphological changes that occur in oocytes, it will be possible to identify critical checkpoints in the reproduction of this endangered species.

## Results and Discussion

Swordfish (*Xiphias gladius)* is a gonochoristic species and females are multiple pelagic spawners with asynchronous ovaries^17–19^. Its oogenesis is similar to those described for other oviparous species with asynchronous development. Hence, by analyzing the morphological features of ovaries, it is possible to detect at the same time the occurrence of follicles at different maturation stages (oogonia, previtellogenic, vitellogenic, mature/hydrated and atretic follicles). Oocyte development is a complex process which involves several biochemical changes leading oogonia to differentiate into mature oocytes ready to be ovulated and then fertilized. During this process, a primary oocyte grows by several orders of magnitude by synthesizing or taking up specific components that will be stored into cortical alveoli, or yolk globules or oil droplets. These components are involved in fertilization process or in the complete development of a new life^1^. To date, information on the macromolecular changes of swordfish oocytes at different developmental stage is lacking. At this purpose in the present study, FTIRI spectroscopy has been applied to get new insights into the macromolecular building of swordfish oocytes at different developmental stages, in terms of composition and topographical distribution of macromolecules of biological interest such as lipids, proteins, carbohydrates and phosphates. A specific focus on cortical alveoli, yolk globules, oil globules and Zona Radiata, has also been outlined. This spectral imaging analysis let map specific areas of non-homogeneous biological samples, generating false color images, that represent the topographical distribution of the total absorption of the infrared radiation. Each pixel corresponds to an IR spectrum. The intensity of the signal associated with a specific IR band provides information both on the amount and the localization within the mapped area of the corresponding molecular/chemical groups.

In fish, oocyte development passes through a first phase of growth (primary growth) followed by a second much more marked one (secondary growth or vitellogenic growth)^20^. Primary growth encompasses the period of oocyte development from oogonia to cortical alveoli stage.

Molecules used at a later stage are directly synthesized from the oocyte itself, and RNA (known as maternal RNA) is accumulated. During this phase, the oocyte remains in meiotic arrest, at the end of prophase until further maturation stage^21^.

**Figure 1:**
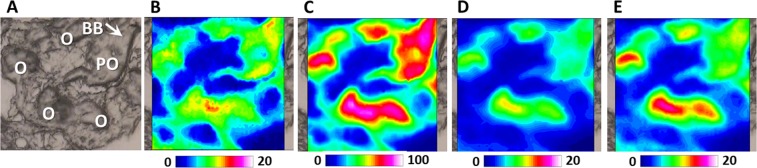
FTIRI analysis of a representative Swordfish ovary section with oogonia (O) and previtellogenic oocytes (PO). (**A**) Microphotograph (328 × 328 μm^2^). IR maps representing the topographical distribution of: (**B**) lipids (LIP), (**C**) proteins (PRT), (**D**) phosphate groups (PHOSPHO), and (**E**) carbohydrates (CARBO). Due to different molar extinction coefficients of the analysed peaks, different scales were used for each IR map (blue colour indicating the areas with the lowest absorption values, while white colour the highest ones). Arrow indicate Balbiani Body (BB).

In Fig. 1A, the microphotograph of an ovarian section at previtellogenic stage, containing oogonia (O) and primary oocytes (PO), is reported. The vibrational imaging analysis shows, both in O and PO, a similar and homogeneous composition of cytoplasm in terms of lipids and proteins, these latter representing the more abundant macromolecules among those investigated (LIP, Fig. 1B, and PRT, Fig. 1C). The analysis of the distribution of lipids and proteins did not highlight respectively the presence of membrane-limited vesicles inside the cytoplasm of the primary oocyte and of the Zona Radiata around it. Conversely, the concomitant accumulation of proteins (PRT, Fig. 1C), phosphates (PHOSPHO, Fig. 1D) and carbohydrates (CARBO, Fig. 1E) in a defined intra-cytoplasmic area of primary oocytes (as indicated by the white arrow in the upper right corner of Fig. 1A) could be probably ascribed to the presence of the Balbiani Body (BB). In vertebrates, the Balbiani Body is asymmetrically positioned generally adjacent to the nucleus of primary oocytes, and it a transient collection of organelles including endoplasmic reticulum, mitochondria, Golgi^22^. In addition, in fish, it contains also RNAs, mainly those encoding germ plasm and patterning proteins^23^.

Primary oocyte growth is mainly due to both cortical alveoli production and deposition of external lipids^24^. Cortical alveoli are membrane-limited vesicles of variable size rich in proteins and carbohydrates synthetized by the oocyte itself. As the oocyte grows, cortical alveoli increase in size and number, filling the ooplasm. The content of cortical alveoli, mainly glycoproteins, is released into the egg surface at the fecundation time as part of the “cortical reaction”^25^.

**Figure 2:**
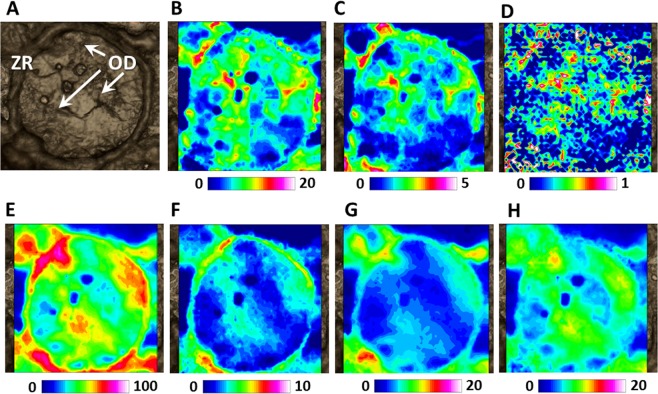
FTIRI analysis of a representative Swordfish oocyte in lipid stage (LS). (**A**) Microphotograph (328 × 328 µm^2^). IR maps representing the topographical distribution of: (**B**) lipids (LIP), (**C**) fatty acids (FA), (**D**) unsaturated lipid alkyl chains (CH), (**E**) proteins (PRT), (**F**) aspartate and glutamate amino acids (COO), (**G**) phosphate groups (PHOSPHO), and (**H**) carbohydrates (CARBO). Due to different molar extinction coefficients of the analysed peaks, different scales were used for each IR map (blue colour indicating the areas with the lowest absorption values, while white colour the highest ones).

In Fig. 2A, a previtellogenic oocyte in lipid stage (LS) is showed. Teleost pelagic eggs are characterized by the presence of several oil droplets which occupy up to half or more of the ooplasm volume^25^. Oil droplets contain mainly neutral lipids rich in monounsaturated fatty acids (FA) that, in fishes, preferentially serve as metabolic energy reserves^25^.

Neutral lipids derive from triglycerides-rich serum lipoproteins. They cross the oolemma either by simple diffusion or via the action of fatty acid transporters or binding proteins and are deposited as droplets in the ooplasm^1^. Synthesis and deposition of neutral lipids is not restricted to primary oocytes and does not end when vitellogenesis and yolk protein deposition are later strongly activated^21^. In the present study, by analysing the false colour images representing the topographical distribution of lipids (LIP, Fig. 2B), fatty acids (FA, Fig. 2C) and unsaturated lipid alkyl chains (CH, Fig. 2D) it was possible to highlight both the crossing of the plasma membrane of unsaturated fatty acids coming from the outer of the cells and their accumulation in the form of oil droplets (OD) within the cytoplasm.

Concomitantly, during lipid stage, the egg envelope named “Zona Radiata” (ZR) begins to form between the oocyte and the surrounding follicular cells^26^. The thickness and complexity of ZR changes gradually during oocyte developmental stages: ZR continues to differentiate throughout the growth of the oocyte becoming highly ordered and architecturally complex during later maturational stages^21^. ZR plays an important role during fertilization. In fact, glycoproteins composing the external part of ZR, have an affinity for spermatozoa and guides a single sperm into the micropyle to reach the egg cell^27^. After fertilization, ZR will protect the embryo in the aquatic environment and will enable gas exchange, excretion and transport of nutrients from the external environment^28^. In the present study, by analysing the false colour images representing the topographical distribution of proteins (PRT, Fig. 2E) and glutamate and aspartate amino acids (COO, Fig. 2F) it was possible to evidence the appearance of a thin ZR.

Finally at this oocyte developmental stage, cytoplasm was characterized by a low amount of phosphate groups (PHOSPHO, Fig. 2G), while carbohydrates (CARBO, Fig. 2H) appear homogeneously distributed.

**Figure 3:**
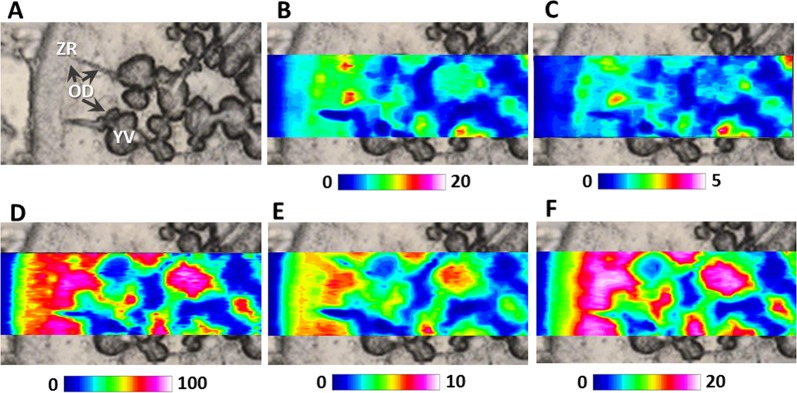
FTIRI analysis of a representative Swordfish vitellogenic oocyte containing the Zona Radiata (ZR), oil droplets (OD) and yolk vesicles (YV). (**A**) Microphotograph (164 × 492 μm^2^). IR maps representing the topographical distribution of: (**B**) lipids (LIP), (**C**) fatty acids (FA), (**D**) proteins (PRT), (**E**) glutamate and aspartate amino acids (COO), and (**F**) carbohydrates (CARBO). Due to different molar extinction coefficients of the analysed peaks, different scales were used for each IR map (blue colour indicating the areas with the lowest absorption values, while white colour the highest ones).

Vitellogenesis is the main event responsible for the huge growing of teleosts oocytes during secondary growth phase^29^. In oviparous vertebrates, vitellogenin (VTG) is a lipoglycophosphoprotein synthetized in the liver and incorporated by the oocyte as major precursor of egg yolk proteins, essential nutrients for future embryogenesis^30^. Once in the ovary, VTG enters the ovarian follicle through capillaries in follicular cells layer. It then passes through the pore canals of the ZR alongside the oocyte microvilli until it makes contact with the oocyte plasma membrane. VTG is incorporated into oocytes by binding specific VTG receptors localized on the oocyte plasma membrane and stored in endosomal vesicles. Finally, VTG containing vesicles blend with lysosome and VTG is cleaved by lysosomal enzymes to generate multiple egg yolk proteins^31^. In the present study, by applying FTIRI spectroscopy, it was possible to characterize at macromolecular level the formation of yolk vesicles from the plasma membrane and their fusion with bigger yolk globules. In Fig. 3A, a portion of a swordfish vitellogenic oocyte (VTG) was showed. The topographic distribution of lipids (LIP, Fig. 3B) and fatty acids (FA, Fig. 3C), let identify in the inner part of the oocyte the concomitant presence of yolk vesicles (YV) and of oil droplets (OD) and outer of the oocyte, the Zona Radiata (ZR). The distribution of proteins (PRT, Fig. 3D), glutamate and aspartate amino acids (COO, Fig. 3E) and carbohydrates (CARBO, Fig. 3F) are evident in both the ZR and YV.

**Figure 4:**
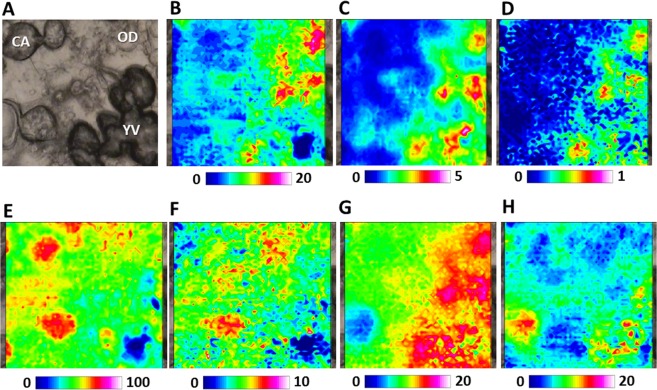
FTIRI analysis of the inner portion of a representative vitellogenic oocyte containing different kinds of structures (cortical alveoli, CA; oil droplets, OD, and yolk vesicles, YV). (**A**) Microphotograph (164 × 164 μm^2^). IR maps representing the topographical distribution of: (**B**) lipids (LIP), (**C**) fatty acids (FA), (**D**) unsaturated lipid alkyl chains (CH), (**E**) proteins (PRT), (**F**) aspartate and glutamate amino acids (COO), (**G**) phosphate groups (PHOSPHO), and (**H**) carbohydrates (CARBO). Due to different molar extinction coefficients of the analysed peaks, different colour scales were used for each IR map (blue colour indicating the areas with the lowest absorption values, while white colour the highest ones).

In Fig. 4A, the inner portion of a vitellogenic oocyte containing at once oil droplets (OD), cortical alveoli (CA) and yolk vesicles (YV) is shown. The occurrence of yolk vesicles (YV) is put well in evidence by the topographical distribution of lipids (LIP, Fig. 4B), fatty acids (FA, Fig. 4C), phosphate groups (PHOSPHO, Fig. 4G,), and, as further extent, also carbohydrates (CARBO, Fig. 4H). Conversely, the presence of oil droplets (OD), rich in fatty acids with a high rate of unsaturation, was monitored by the distribution of fatty acids (FA, Fig. 4C) and unsaturated lipid alkyl chains (CH, Fig. 4D). Cortical alveoli (CA), mainly composed by glycosylated proteins and poor in lipids, were highlighted by the topographical distribution of proteins (PRT, Fig. 4E), glutamate and aspartate amino acids (COO, Fig. 4F), and also carbohydrates (CARBO, Fig. 4H).

**Figure 5:**
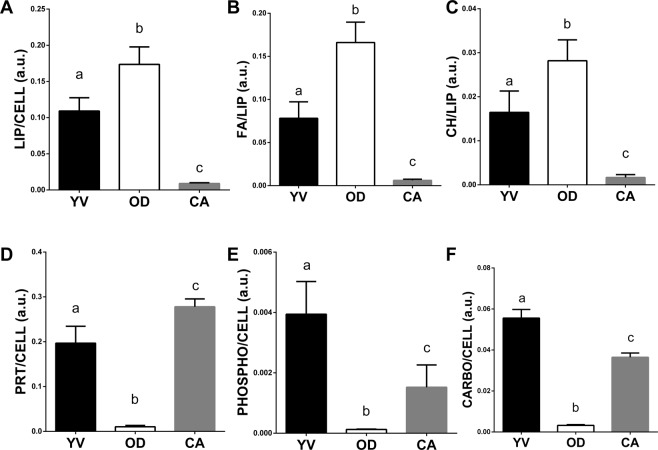
Macromolecular composition of yolk vesicles (YV), oil droplets (OD) and cortical alveoli (CA) into vitellogenic oocytes of swordfish species. Univariate analysis of the following band area ratios: (**A**) LIP/CELL, (**B**) FA/LIP, (**C**) CH/LIP, (**D**) PRT/CELL, (**E**) PHOSPHO/CELL, and (**F**) CARBO/CELL. Different letters indicate statistically significant differences among experimental groups (p < 0.05).

The macromolecular characterization of yolk vesicles (YV), oil droplets (OD) and cortical alveoli (CA) into vitellogenic oocytes was also obtained by a semiquantitative analysis of the spectral data (Fig. 5). Yolk vesicles (YV) resulted rich in proteins (PRT/CELL), phosphate groups (PHOSPHO/CELL), and carbohydrates (CARBO/CELL). Cortical alveoli (CA) contained above all proteins (PRT/CELL), carbohydrates (CARBO/CELL) and phosphate groups (PHOSPHO/CELL), while they were poor in lipids (LIP/CELL) and fatty acids (FA/LIP). Finally, oil droplets (OD) were found to be characterized by a high concentration of lipids (LIP/CELL) and, mainly, fatty acids (FA/LIP) with a high rate of unsaturation level (CH/LIP), and a really low amount of phosphate groups (PHOSPHO/CELL), proteins (PRT/CELL) and carbohydrates (CARBO/CELL).

**Figure 6:**
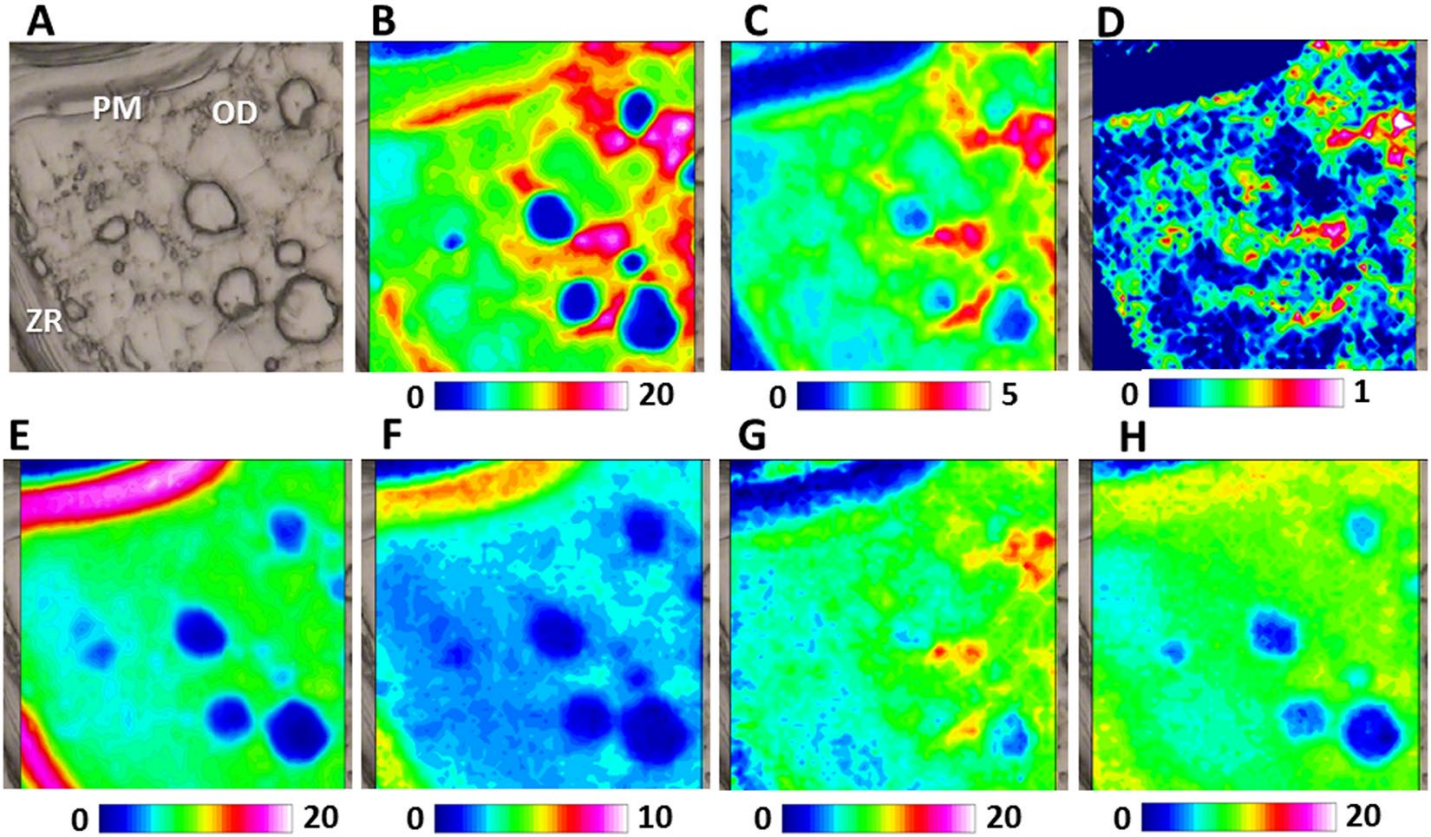
FTIRI analysis of a portion of a representative mature oocyte (MAT), containing the Zona Radiata (ZR), the plasma membrane (PM) and oil droplets (OD). (**A**) Microphotograph (164 × 164 μm^2^). IR maps representing the topographical distribution of: (**B**) lipids (LIP), (**C**) fatty acids (FA), (**D**) unsaturated lipid alkyl chains (CH), (**E**) proteins (PRT), (**F**) aspartate and glutamate amino acids (COO), (**G**) phosphate groups (PHOSPHO), and (**H**) carbohydrates CARBO). Due to different molar extinction coefficients of the analysed peaks, different scales were used for each IR map (blue colour indicating the areas with the lowest absorption values, while white colour the highest ones).

A portion of a hydrated mature oocyte (MAT) is reported in Fig. 6A. The cytoplasm appears homogeneous and not organized in vesicles; the presence of holes could be attributed to the high degree of hydration typical of pelagic eggs. Following the distribution of lipids (LIP, Fig. 6B), it is possible to evidence the plasma membrane (PM). The occurrence of oil droplets (OD) which do not appear still fused in a central oil globule, is well highlighted by the distribution of unsaturated fatty acids (indicated by the concomitant distribution of both fatty acids, FA, Fig. 6C, and unsaturated alkyl chains CH, Fig. 6D). In this stage, it is well evident the increase of the Zona Radiata, rich in glycosylated proteins (PRT, Fig. 6E; COO, Fig. 6F, and CARBO, Fig. 6H). Conversely, phosphate groups (PHOSPHO, Fig. 6G) and unsaturated fatty acids (FA, Fig. 6C, and CH, Fig. 6D) are not detected in this area.

**Figure 7:**
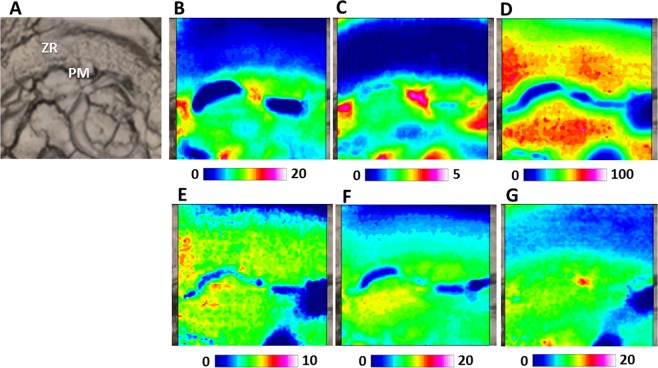
FTIRI analysis of portion of a representative atretic oocyte (ATR), containing the Zona radiata (ZR) and the plasma membrane (PM). (**A**) Microphotograph (164 × 164 μm). IR maps representing the topographical distribution of: (**B**) lipids (LIP), (**C**) fatty acids (FA), (**D**) proteins (PRT), (**E**) aspartate and glutamate amino acids (COO), (**F)** phosphate groups (PHOSPHO), and (**G**) carbohydrates (CARBO). Due to different molar extinction coefficients of the analysed peaks, different scales were used for each IR map (blue colour indicating the areas with the lowest absorption values, while white colour the highest ones).

A portion of an atretic oocyte (ATR) is reported in Fig. 7A. Ovarian atresia is a common phenomenon in vertebrate ovaries during which a number of ovarian follicles recruited into the vitellogenesis pool fail to complete maturation and ovulation^1^. The process of atresia and resorption of ovarian follicles in fish is characterized by oocyte marked morphological changes. The first morphological signs of atresia is the disintegration of cytoplasmic organelles (mitochondria, cortical alveoli, ...), followed by the fragmentation of the Zona Radiata^1^. By comparing FTIRI results achieved on vitellogenic and atretic oocytes it was possible to obtain an imaging molecular signature that could readily and reliably differentiate vitellogenic oocytes from atretic ones. In particular, the Zona Radiata of the atretic oocyte shows a very different composition and organization with respect to that of a vitellogenic one. In particular, by analysing the IR map, he total absence of lipids (LIP, Fig. 7B, and FA, Fig. 7C), and lower amounts of proteins (PRT, Fig. 7D), glutamate and aspartate amino acids (COO, Fig. 7E), phosphate groups (PHOSPHO, Fig. 7F) and carbohydrates (CARBO, Fig. 7G) is observed. In addition, the plasma membrane (PM) is differently organized as indicated by the distribution of lipids (LIP, Fig. 7B), and cytoplasm is not homogeneous and presents a great number of vacuoles.

**Figure 8:**
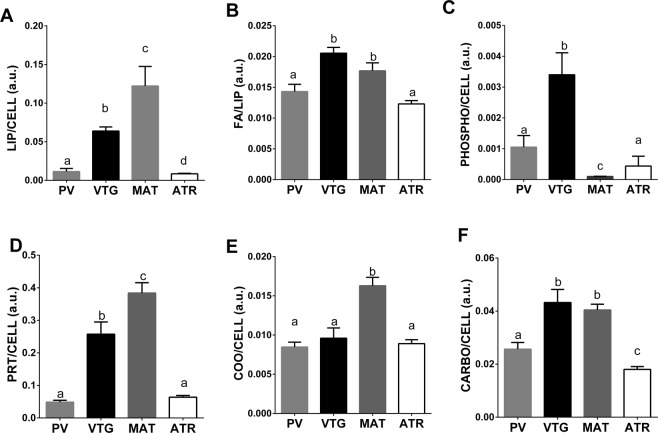
Macromolecular composition of the Zona Radiata of previtellogenic (PV), vitellogenic (VTG), mature (MAT) and atretic (ATR) oocytes of swordfish species. Univariate analysis of the following band area ratios: (**A**) LIP/CELL, (**B**) FA/LIP, (**C**) PHOSPHO/CELL, (**D**) PRT/CELL, (**E**) COO/CELL, and (**F**) CARBO/CELL. Different letters indicate statistically significant differences among experimental groups (p < 0.05).

A deeper focus on ZR macromolecular composition was done by applying a spettroscopic semiquantitative analysis comparing ZR from previtellogenic (PV), vitellogenic (VTG), mature (MAT) and atretic (ATR) oocytes (Fig. 8). Nara *et al*. already reported an FTIR study on the changes occurring in protein structure of Zona Radiata of mammal oocytes during fertilization^12^. Nevertheless, in the present study, the coupling of spectral imaging with semiquantitative analysis of IR data let highlight, for the first time, the modifications in the macromolecular composition of Zona Radiata of oocytes at different developmental stage. In particular, ZR from vitellogenic (VTG) and mature (MAT) oocytes showed with respect to that from oocytes in previtellogenic phase, a higher level of proteins (PRT/CELL), aspartate and glutamate amino acids (COO/CELL) and carbohydrates (CARBO/CELL), lipids (LIPIDS/CELL) and fatty acids (FA/LIP). A significant higher amount of phosphate groups (PHOSPHO/CELL) was observed only in the Zona Radiata of vitellogenic oocytes (VTG). The presence of lipid-containing compounds within the ZR may be ascribable to the vitellogenin crossing of the numerous canal pore of ZR directly connecting oocytes with surrounding follicular cells^32^. Once again, the Zona Radiata of atretic oocytes (ATR) shows a peculiar macromolecular trait, completely different from vitellogenic and mature oocyte and more similar to the previtellogenic one. It results characterized by very low levels of phosphate groups (PHOSPHO/CELL) and fatty acids (FA/LIP).

Concluding, the present study represents significant progress in the comprehensive understanding of swordfish oogenesis process. The spectral characterization of swordfish oocytes at different developmental stages is a starting point and a useful tool to evaluate changes on egg quality related to different conditions. Further studies are in progress with the aim to evaluate egg composition modifications in females caught in different periods of the year or in different fishing area (within the Mediterranean sea as well as in the Atlantic Ocean) in order to have a clearer picture of reproductive performance of swordfish. These results will be of great importance to support the International Commission for the Conservation of Atlantic Tunas (ICCAT) in the optimization of the recovery plan for Mediterranean swordfish adopted from 2016.

## Methods

### Sample preparation

10 swordfish (*Xiphias gladius)* females with a Lower Jaw-Fork Length (LJFL) >100 cm (according to Italian legislation) were caught by commercial vessels using long lines in the period May–July 2017, in the central Mediterranean Sea (Sardinia and Sicily). The fish were caught for commercial purpose and ovaries samples were collected under the guidelines of the biological samples indicated in the ICCAT manual. The procedures did not include animal experimentation, and ethics approval is not necessary in accordance with the Italian legislation (D.L. 4 of Mars 2014, n. 26, art. 2). Soon after capture, ovaries were removed; a gonad portion (∼2 cm^3^) was picked up from the middle part of the ovary of all specimens and preserved at −80 °C for FTIRI analysis.

### FTIRI measurements and data analysis

From each frozen ovarian sample, three thin sections (∼10 μm thickness) were cut at 200 μm from each other, by using a cryomicrotome. Sample sections were then deposited, without any fixation process, onto CaF_2_ optical windows (1 mm thickness, 13 mm diameter) and air-dried for 30 min. FTIRI measurements were performed within 48 hours after cutting at the Infrared Beamline SISSI (Synchrotron Infrared Source for Spectroscopy and Imaging), Elettra Sincrotrone Trieste (Trieste, Italy). This procedure was already carried out on similar samples and a good stability in terms of infrared features was always observed^33^. A Bruker VERTEX 70 interferometer coupled with a Hyperion 3000 Vis-IR microscope was used. The spectrometer was equipped with a liquid nitrogen cooled bidimensional Focal Plane Array (FPA) detector that allows to perform the imaging analysis of non-homogeneous biological samples by simultaneously acquiring 4096 spectra on an area of 164 × 164 μm^2^. The visible image of each ovarian section was obtained with a 15X condenser/objective and used to select areas containing oocytes at different development stages (previtellogenic, vitellogenic, mature, and atretic). On these selected areas, IR maps were collected in transmission mode in the 4000–900 cm^−1^ MIR range with a spatial resolution of ~2.56 μm. Each spectrum was the result of 256 scans with a spectral resolution of 4 cm^−1^. The size of the areas to be mapped was chosen based on oocytes’ dimension. Background spectra were acquired on clean regions of CaF_2_ optical windows.

Raw IR maps were corrected by applying the Atmospheric Compensation routine, to remove the contribution of atmospheric carbon dioxide and water vapour, and then vector normalized in the 4000–900 cm^−1^ spectral range, to avoid artefacts due to differences in thickness (OPUS 7.1 software, Bruker Optics, Ettlingen, Germany).

These preprocessed IR maps were integrated under the following spectral regions, to obtain false colour images representing the topographical distribution and relative amount of the most relevant biochemical features^34^: 3034–2995 cm^−1^ (containing the vibrational modes of unsaturated groups in lipid alkyl chains, named CH); 2995–2825 cm^−1^ (containing the vibrational modes of lipids, named LIP); 1754–1718 cm^−1^ (containing the vibrational modes of fatty acids, named FA); 1718–1481 cm^−1^ (containing the vibrational modes of proteins, named PRT); 1427–1360 cm^−1^ (containing the vibrational modes of COO^−^ groups in glutamate and aspartate amino acids, named COO); 1274–1181 cm^−1^ (containing the vibrational modes of phosphates groups inside nucleic acids, named PHOSPHO), and 1130–1013 cm^−1^ (containing above all the vibrational modes of carbohydrates, named CARBO). An arbitrary colour scale was used, white colour indicating areas with the highest absorbance values and blue colour areas with the lowest ones.

For a deeper analysis of the cellular compartments of oocytes at different development stages (oogonia (O) primary oocyte, PO; lipid stage, LS; vitellogenic, VTG; mature/hydrated, MAT, and atretic, ATR), some microareas representative of yolk vesicles (YV), lipid droplets (LD) and Zona Radiata (ZR) were chosen, each containing at least 200 IR spectra. These IR spectra were integrated under the same spectral regions above defined (Integration routine; OPUS 7.1 software package, Bruker Optics, Ettlingen, Germany). The sum of the integrated areas 3050–2800 and 1770–950 cm^−1^ was considered indicative of the total cell biomass (CELL). Integral values were used to calculate specific band area ratios (reported in the Results section).
